# The corticosteroids effect on corneal endothelial cell in pulse therapy, specific to the cataract surgery

**Published:** 2014

**Authors:** AC Ghita, AM Ghita, M Noaghi, A Popa Cherecheanu

**Affiliations:** * Center for Excellence în Neuroscience, Department of Functional Sciences – Division of Physiology and Neuroscience, “Carol Davila” University of Medicine and Pharmacy, Bucharest, Romania, 8 Eroii Sanitari Blvd, 050474 Bucharest, Romania; ** Division of Physiology, “Carol Davila” University of Medicine and Pharmacy, Bucharest, Romania, 8 Eroii Sanitari Blvd, 050474 Bucharest, Romania; *** University Emergency Hospital Bucharest, Sector 5, 169 Independentei Blvd, Bucharest, Romania

**Keywords:** corticosteroids, cataract, cataract surgery, endothelial cell

## Abstract

Rationale: We suspect a protective role of corticosteroids in pulse therapy in cataract surgery

Objective: The monitoring of the corticosteroids effect in pulse therapy after cataract surgery, associated with conventional therapy drugs on endothelial cells.

Methods: According to the effective phacoemulsification time (EPT), we have created a lot with hard cataract (EPT>10s) and a lot with soft cataract (EPT<10s). Each lot had a control group treated with local steroids and a study group treated with local steroids to which steroids in pulse therapy were associated postoperative for 3 days.

Results We noticed a smaller loss of endothelial cell in the study group with soft cataract compared to the control group but without statistic significance. In the study group with hard cataract the recovery of the visual function is faster than in the control group. The loss of endothelial cell compared to the EPT is similar at one week in both of the examined groups in patients with hard cataract (39.1±13.2 cells/mm2/s study group and 41.51±18.5 cells/mm2/s control group). In the 1 month postoperative examination, we could find a significantly bigger loss in the control group (40.18 ±16.6 cells/mm2/s) compared to the study group (24.48±7 cells/mm2/s) (p<0.05).

Conclusions: The administration of corticosteroids in pulse therapy associated to topic therapy seems to be benefic in the limitation of the loss of endothelial cell specific to cataract surgery. The major benefit of pulse therapy appears in patients with hard cataract and in patients with a lower endothelial reserve in whom surgery is mandatory.

## Introduction

Cataract represents a transparency disorder of the lens characterized by opacification. The most frequent cause of cataract is due to ageing processes and in addition to that, smoking, radiation exposure, nutritional and toxic factors, high blood pressure, metabolic diseases [**1**-**4**]. The new technology of ophthalmological microsurgery facilitates the intervention for cataract, visual acuity being regained by extraction of the opacified lens and implantation of an artificial intraocular lens. [**5**]. In the last century the progresses made in cataract surgery are not accompanied by progresses in understanding the formation of cataract, means of prevention and non-surgical treatment. [**3**;**4**] 

During and after cataract surgery different complications could appear. The most common are: posterior capsule rupture during surgery and intraocular inflammation, cystoids macular edema as postoperative incidents. [**6**] However, postoperative corneal edema due to loss of endothelial cell density and dysfunction of the pumps in the remaining endothelial cell is one of the most severe complications that appears after any type of lens extraction, with a global incidence of 1%. [**7**.**8**].The amount of endothelial cell decreases naturally with age [**9**], but to this physiologically loss, the negative effect of the lens extraction is added. [**7**;**10**-**11**]

Stromal corneal edema appears as a consequence of endothelial cell density loss [**12**] and due to the malfunction of the endothelial pump, whose role would be to maintain the stroma and the epithelial layer in a relatively state of dehydration. An increase in intraocular pressure [**13**;**14**] and inflammation may determine the malfunction of the ionic pumps [**13**;**15**;**16**], meanwhile, a decrease in intraocular pressure and anti-inflammatory medication may reduce the edema and re-establish the corneal clarity. [**16**] Conventional postoperative therapy with topical steroid and non-steroid anti-inflammatory is proven to be of great help in maximization the percentage of the remaining endothelial cell s, therefore the optimization of regaining corneal transparency. In practice, topical steroids (prednisolone acetate 1% or dexamethasone sodium phosphate 0.1%) are used frequently in postoperative corneal edema. [**17**;**18**] However, conventional therapy with topical steroids and non-steroids won’t prevent always the development of epithelial bullous keratopathy, this is the reason why is necessary to discover new therapies and alternative treatments to diminish the rate of endothelial cell loss in high risk cases.

The purpose of this study is to establish the efficiency of an alternative therapy in hard cataracts with a high risk of severe postoperative complications such as corneal decompensation and epithelial bullous keratopathy. So, we evaluated the efficiency and the risks of corticotherapy with methylprednisolone administered in pulse therapy and associated with conventional therapy.

## Materials and methods

The study was performed on a group of 30 patients, analyzing 32 eyes with cataract. Patients were investigated, surgically treated and monitored in the Ophthalmology Clinic of the Emergency University Hospital, Bucharest. Supplementary paraclinical examinations were performed in the private Ophthalmology West Eye Hospital. The dates from this observational study were collected in a prospective manner between October 2012- June 2013. The study respects the ethical criteria of the medical community, provided in the WHA Helsinki Declaration. 

Patients were divided in two lots: one group of 16 patients with hard cataract with an EPT (effective phacoemulsification time) greater than 10 seconds and the other group, also of 16 patients, with soft cataract and EPT under 10 seconds. Every group was then divided in two subgroups: the control group with topical steroid therapy, and the study group with topical and systemic steroid therapy. Therefore, we actually have four groups: the study group with hard cataract (6 patients), the study group with soft cataract (7 patients), the control group with hard cataract (10 patients) and the control group with soft cataract (9 patients). Patients had one admission in the hospital during the surgery and two ambulatory follow-ups at 1 week and then at 1 month after surgery.

In the study were included patients with cataract diagnosis confirmed at slit-lamp examination, that signed the informed consent for cataract surgery, after they were explained the procedure and the possible complications. Were excluded from the study, the patients incapable of understanding the procedure, patients with mental illness or those who refused treatment or the tests necessary for follow-up, patients with ocular trauma or intraocular surgery in antecedents, those with co-existing ocular disorders (infections or inflammation of the eye, insufficient pupillary dilatation during surgery, intraoperative complications such as posterior capsule rupture, systemic disease that represent contraindications for corticosteroid therapy, uncooperative patients and those that missed the post-surgical mandatory follow-up.

Cataract surgery and the perioperative and postoperative therapy

All the patients were examined and treated surgically by the same ophthalmologist using the same equipment and the same surgical technique (stop and chop phacoemulsification and foldable artificial intraocular lens). For all the patients retrobulbar anesthesia was performed and were taking into account the intraoperative phacoemulsification characteristic parameters APT (average phacoemulsification time) and EPT. The therapy in control groups was initiated starting with day-one after surgery: dexamethasone 0.1% topical, 4 times a day, for 3 weeks with gradually decrease in the last week. In the study groups was administered methylprednisolone 500mg/day (Solu-Medrol) for 3 days, starting in the surgery day and dexamethasone 0.1% topical, 4 times a day, for 3 weeks starting day-one after surgery and with gradually decrease in the last week. In all fourth groups it was administered in the same time with the dexamethasone 0.1% also a topical non-steroid anti-inflammatory.

Clinical and paraclinical exam

One day before surgery, at 1 week and 1 month after surgery the following clinical and paraclinical parameters were performed at each visit: VA (visual acuity) for BE (both eyes), visual field, IOP (intraocular pressure), CCT (central corneal thickness), endothelial cell density and slit-lamp examination.

The visual acuity was evaluated using Snellen scale and the intraocular pressure was measured using Goldmann aplanotonometry. Visual acuity is used as a parameter which subdivides all patients in four subgroups: patients with good visual acuity (greater than 0.5), patients with mild decrease of visual acuity (less or equal with 0.5), patients with low visual acuity (less or equal with 0.25) and patients with very low visual acuity (lesser than 0.125).

Specular microscopy was used to observe and to record non-invasively the endothelial cell s. Both corneal central thickness and endothelial cell density were obtained by measuring the center of the cornea using a non-contact specular microscope SP3000-P® (Topcon Corporation, Japan). The determinations were made the day before surgery, at 1 week and at 1 month after cataract surgery.

Statistical data analysis

The results were presented as a median +/- standard deviation. The data were introduced into tables using Microsoft Excel 2010, exported and analyzed using IBM SPSS Statistics 2 and using also the general elements of descriptive statistics for the analyzed variables (graphic representation). The statistic tests used were: t-student for independent samples and the One Way Anova Test. A level of p <0.05 was accepted as statistically significant.

## Results

**Fig. 1:**
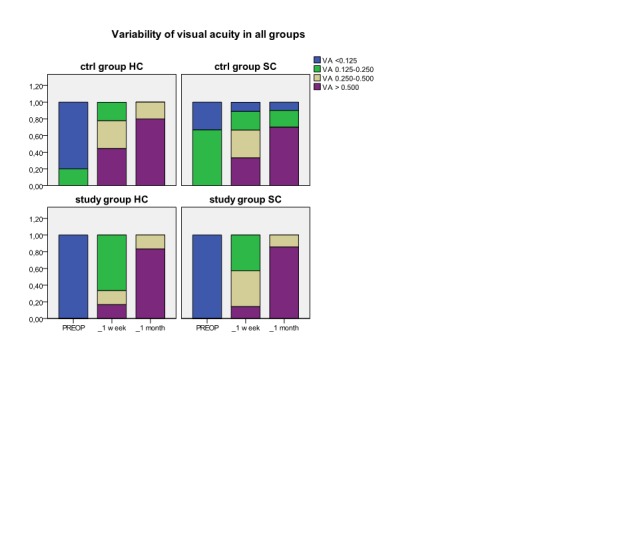
Variability of visual acuity (VA) in each group preoperative, at 1 week and 1 month postoperative in hard cataract (HC) and in soft cataract (SC)

The administration of corticosteroids in pulse therapy associated to conventional therapy from cataract surgery didn’t have major side effects. Although, for reducing the eventual risks that could appear, we eliminated from the group the diabetic patients and another risk categories and we also administrated to the patients protons pump inhibitors in association with a specific diet. Watching visual acuity, we observed that surgical intervention brings a good postoperative visual outcome in all four groups that we have examined, with a faster recovery in patients with soft cataract and slower in patients with hard cataract. (**Fig. 1**). After the cataract surgery, the intraocular pressure was in normal ranges with minimal variation postoperative. No statistical *significant differences* existed between studied groups preoperative and postoperative at one week and one month. We measured the mean central corneal thickness preoperative in each examined group and we didn’t find any *statistical significant differences between them*.

In hard cataract group there weren’t *statistical significant differences* between average EPT from the control group (4.77±1.68 s) and the average EPT from the study group (19.71±5.46s). Because of the differences between EPT in hard cataract group, we compared the changes in cell density in absolute values and also in values related to EPT.

**Fig. 2:**
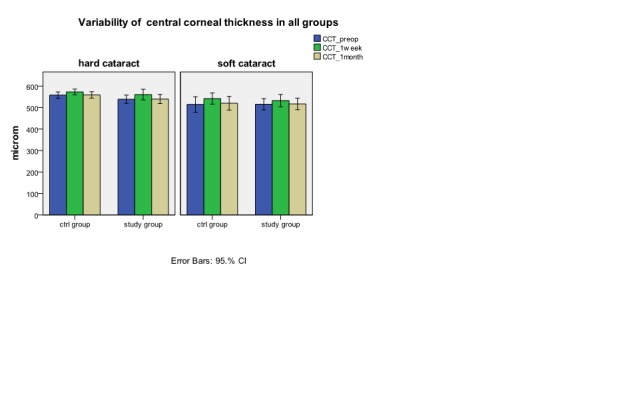
Variability of CCT (central corneal thickness) in all four groups preoperative, at 1 week and 1 month postoperative (median ± error bars) in hard cataract (HC) and in soft cataract (SC)

**Fig. 3:**
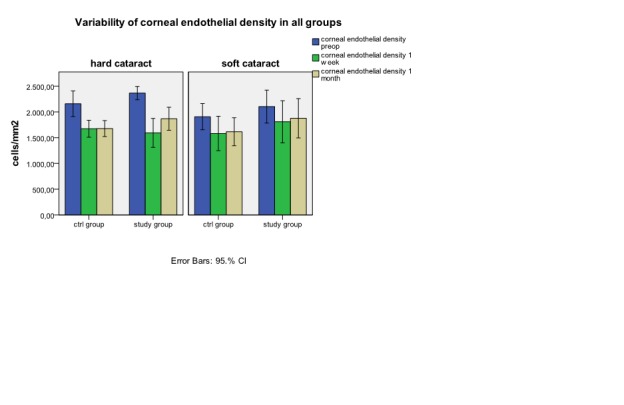
Variability of corneal endothelial density in each group preoperative, at 1 week and 1 month postoperative (median ± error bars).

After cataract surgery, the mean central corneal thickness raised in all examined groups at one week and returned to initial values at one month (**Fig. 2**) *without statistical difference*. We watched the corneal endothelial density separate for each group. As a general observation, in all groups there is a decrease of endothelial cell density in the absolute value at the postoperative follow up.(**Fig. 3**) The decrease is important in all groups at one week with a partial recovery at one month postoperative. The hard cataract group has a bigger lost in endothelial cell density (p<0.05) compared to soft cataract group at one week (592.75±270.82 cells/mm2 versus 312.31±284.2 cells/mm2) and one month postoperative (487.43±190.38 cells/mm2 versus 263±204.1 cells/mm2).

**Fig. 4 a) and 4b):**
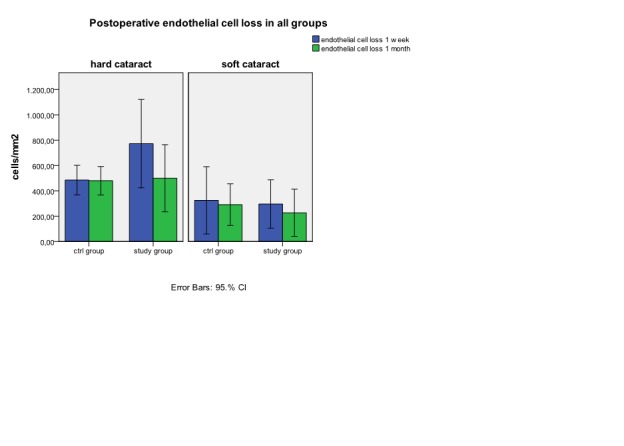
Postoperative endothelial cell lost in absolute values in hard cataract (a) and in soft cataract (b)

**Fig. 5:**
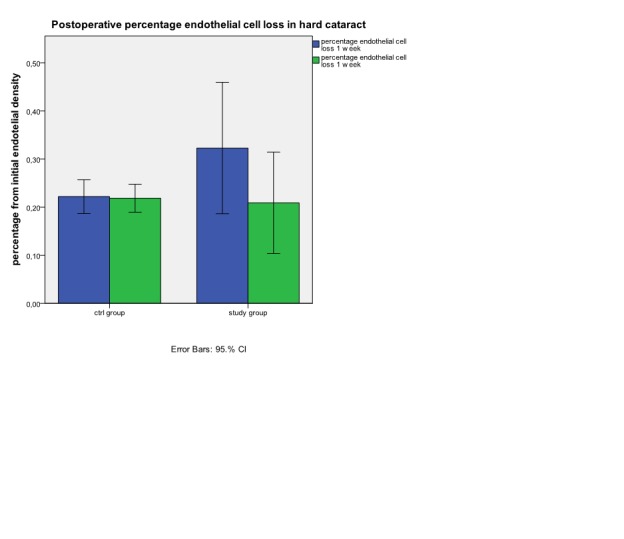
Postoperative percentage endothelial cell loss in hard cataract at 1 week and 1 month

*In hard cataract group the absolute values (**Fig. 4a**) and the percentage values (**Fig. 5**) show an important decrease in endothelial cell density from initial values at one week (p<0.05) in patients from the study group (772.33±332.7 cells/mm2) versus control group (485.5±162.4 cells/mm2). At one month we can observe a good recovery of endothelial density in study group , with a final mean lost* of 499.5±252.9 cells/mm2 . This value is similarly with the final mean lost in control group (480.2±157.2 cells/mm2). 

**Fig. 6a) and 6b):**
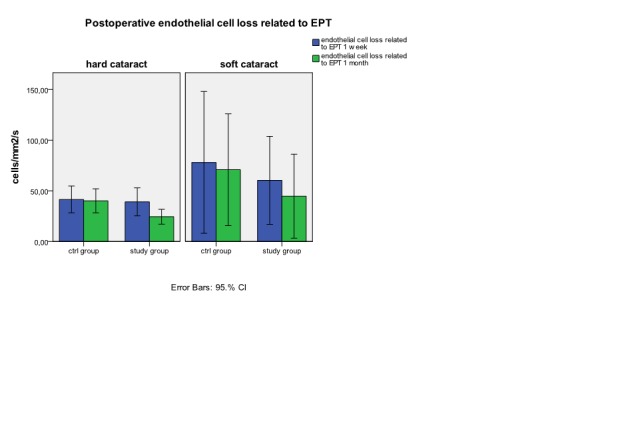
Postoperative endothelial cell loss related to EPT in hard cataract (a) and in soft cataract (b)

Comparing the loss of endothelial cell density in soft cataract lot in absolute value (**Fig. 4b**), and reported to EPT (**Fig. 6b**) we observe a higher lost at one week as at one month in control group (325.22 ±345.14 cells/mm2 versus 291±214.19 cells/mm2) compared to study group (295.71± 206.17cells/mm2 versus 227±200.74 cells/mm2) but with any *statistical significant difference*. 

**Fig. 7:**
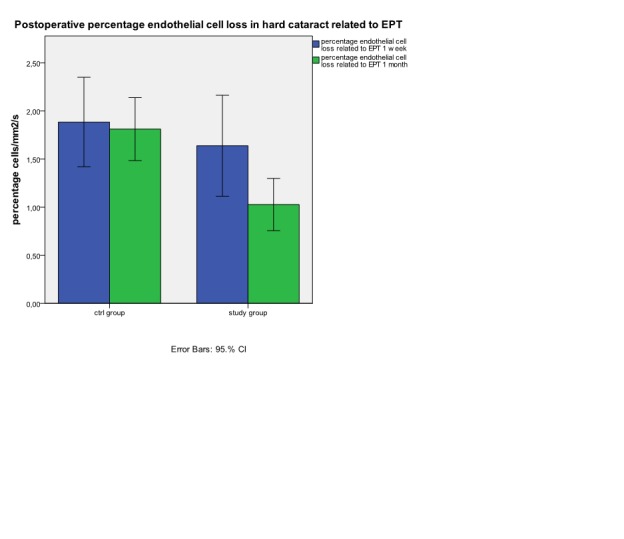
Postoperative percentage endothelial cells loss in hard cataract related to EPT at 1 week and 1 month

If in exchange, in hard cataract lot we relate the loss of endothelial cell to the EPT (**Fig. 6a**) we can observe at one week a similar lost in endothelial cell density in both examined groups (39.1±13.2 cells/mm2/s versus 41.51±18.5cells/mm2/s).Similar values at one week we can find for percentage loss of endothelial cell density from the initial value related to EPT (1.63±0.5%/s in study group versus 1.88±0.5%/s in control group). At one month, endothelial cell density loss related to EPT is significant lower in study group (24.48±7 cells/mm2/s) versus control group (40.18±16.6 cells/mm2/s). The result at 1 month can be also observed by comparing the percentage loss of endothelial cell density related to EPT (1.02±0.3%/s study group versus 1.81±0.5%/s in control group). (**Fig. 7**) 

## Discussions

Cataract is the principal leading cause of blindness who has an efficient treatment.[**1**;**19**] Once with the major progresses made in the technology field, trough cataract surgery beside removing the opacified lens, the target is to obtain the best visual acuity being minimally invasive and by having a certain degree of safety. [**20**;**21**] One of the problems to whom cataract surgeons are confronting with, after extraction of cataract and implantation of intraocular lens, is corneal decompensation because of endothelial cell density loss. Surgical procedure in every cases leads to a decrease in the number of endothelial cell but this lost of cells is secondary to a certain risk factors. At the moment, we don’t have a standard procedure to follow for decreasing the loss of endothelial cell in the postoperative period, so further studies are needed. Patients from all the groups have the same distribution of the clinical type of cataract and age groups similar with specialty literature. The patients have ages between 52 and 81 years old, with a mean of 68 years old (standard deviation ±9 years). The distribution results on age groups of analyzed patients are in agreement according to Beaver Dam Eye Study. [**19**;**22**]

The visual acuity of patients from control groups, as from study groups has significant improvement one week after cataract surgery and the recovery of visual function continues one month after the surgery in all study groups. During cataract surgery corneal endothelial cell are affected in certain degrees which is connected with the type of surgical technique used [**11**;**23**;**24**], this is the reason why all the patients included in the study were operated by the same cataract surgeon using the same surgical technique. There is a relation between the total phacoemulsification time and effective time and the loss of corneal endothelial cell [**10**;**23**]. First of all, phacoemulsification affects the cornea trough destruction of the corneal endothelial cells [**25**]. An important factor associated to surgery, is inflammation which determines loss of endothelial cell and changes in endothelial ionic pumps which can cause a certain degree of endothelial dysfunction [**17**;**26**]. This is the reason why local anti-inflammatory non-steroidal and steroidal drugs are needed. Watching soft cataract group we can observe a favorable evolution with a reduced loss of endothelial cell but without any statistic significance. The percentage values of the degree of endothelial dysfunction at one month obtained in this study are in soft cataract group 11.43% - 15.29% and 20.9% - 21.9% in hard cataract group. These results are according to those from specialty literature with a loss of endothelial cell trough phacoemulsification between 8.5-15.4% [**25**;**27**]. Our results show that in hard cataract, where surgical trauma is bigger, the inflammatory postoperative phenomena are higher. So, the administration of corticosteroids in pulse therapy is helpful in obtaining the maximal number of endothelial cell at one month (24.481cells/mm2/s in the study group versus 40.18/cells/mm2/s in the control group; p<0.05). Taking into account that endothelial cell loss, in its absolute value, is higher in hard cataract group (592.75 cells/mm2 at one week versus 487.44 cells/mm2 at one month ) the associations of general corticosteroid therapy to the conventional treatment has a bigger chance to restore the corneal clarity. According to literature dates, the corticosteroids stimulate Na+/K+ ATP-ase and the ionic pumps from the corneal endothelium [**26**;**27**]. Although at one week postoperative, the effects of pulse therapy combined to local therapy aren’t superior to those from control group, final recovery of the endothelium under systemic corticosteroid is significant higher at one month.

Pachymetry, although confirms the corneal damage during cataract surgery, isn’t a reliable parameter of endothelial cell lost. Pachymetry being more connected with endothelial pump dysfunction [**24**;**26**]. A raise of central corneal thickness at one week shows that endothelial dysfunction is still present and that will disappear at one month despite endothelial cell lost. The study limits are a small number of patients included in analyzed groups which leads to inappropriate statistic results and a short period of follow-up. So, we cannot establish for how long the effect of corticosteroids administrated in pulse therapy is benefic on diminishing endothelial cell lost. And we cannot monitories on long term if any possible complications may appear.

## Conclusions

Phacoemulsification affects the cornea trough loosing and dysfunction of endothelial cells. The degree of corneal involvement goes directly proportional with the intensity of ultrasounds used. Corticosteroids in pulse therapy associated with topical therapy seems to have benefits in reducing the amount of endothelial cells that can be lost after cataract surgery. In soft cataract (EPT<10s) the benefits are minimal compared with potential side effects of corticosteroids pulse therapy although in hard cataract where there is an important trauma associated with high phacoemulsification time (EPT>10s), the corticosteroids in pulse therapy associated to the topical therapy lead to a good control of intraocular inflammation after cataract surgery. The major benefit after corticosteroids pulse therapy appears at patients with hard cataract and at patients with a reduced number of endothelial cells before cataract surgery.

**No financial interest.**

## References

[1] Laitinen A, Laatikainen L, Harkanen T, Koskinen S, Reunanen A, Aromaa A (2010). Prevalence of major eye diseases and causes of visual impairment în the adult Finnish population: a nationwide population-based survey. Acta Ophthalmol.

[2] Vashist P, Talwar B, Gogoi M, Maraini G, Camparini M, Ravindran R.D., Murthy G.V., Fitzpatrick K.E., John N, Chakravarthy U, Ravilla T.D., Fletcher A.E. (2011). Prevalence of cataract în an older population în India: the India study of age-related eye disease. Ophthalmology.

[3] Gupta V.B., Rajagopala M, Ravishankar B (2013). Etiopathogenesis of cataract: An appraisal. Indian J. Ophthalmol.

[4] Klein B.E., Klein R, Jensen S.C., Linton K.L. (1995). Hypertension and lens opacities from the Beaver Dam Eye Study. Am. J. Ophthalmol.

[5] Gomez M.L. (2014). Measuring the quality of vision after cataract surgery. Curr. Opin. Ophthalmol.

[6] McKellar M.J., Elder M.J. (2011). The earl complications of cataract surgery: is routine review of patients 1 week after cataract extraction necessary?. Ophthalmology.

[7] Kosrirukvongs P, Slade S.G., Berkeley R.G. (1997). Corneal endothelial changes after divide and conquer versus chip and flip phacoemulsification. J. Cataract Refract. Surg.

[8] Liesegang T.J. (1991). The response of the corneal endothelium to intraocular surgery. Refract. Corneal Surg.

[9] Laule A, Cable M.K., Hoffman C.E., Hanna C (1978). Endothelial cell population changes of human cornea during life. Arch. Ophthalmol.

[10] Mahdy M.A., Eid M.Z., Mohammed M.A., Hafez A, Bhatia J (2012). Relationship between endothelial cell loss and microcoaxial phacoemulsification parameters în noncomplicated cataract surgery. Clin. Ophthalmol.

[11] Park J, Yum H.R., Kim M.S., Harrison A.R., Kim E.C. ((2013). Comparison of phaco-chop, divide-and-conquer, and stop-and-chop phaco techniques în microincision coaxial cataract surgery. J. Cataract Refract. Surg.

[12] Goncalves E.D., Campos M, Paris F, Gomes J.A., Farias C.C. (2008). Bullous keratopathy: etiopathogenesis and treatment. Arq Bras. Oftalmol.

[13] Gagnon M.M., Boisjoly H.M, Brunette I, Charest M, Amyot M (1997). Corneal endothelial cell density în glaucoma. Cornea.

[14] Novak-Stroligo M, peza-Dunato Z, Kovacevic D, Caljkusic-Mance T (2010). Specular microscopy în glaucoma patients. Specular microscopy în glaucoma patients.

[15] O'Brien W.J., Palmer M.L, Guy J, Taylor J.L. (1996). Endothelial barrier function and Na+/K(+)-ATPase pump density în herpetic stromal disease. Invest Ophthalmol. Vis. Sci.

[16] Macdonald J.M, Geroski D.H., Edelhauser H.F. (1987). Effect of inflammation on the corneal endothelial pump and barrier. Curr. Eye Res.

[17] Hatou S, Yamada M, Mochizuki H, Shiraishi A, Joko T, Nishida T (2009). The effects of dexamethasone on the Na,K-ATPase activity and pump function of corneal endothelial cell s. Curr. Eye Res.

[18] Nissen J.N., Ehlers N, Frost-Larsen K, Sorensen T (1993). The effect of topical steroid on postoperative corneal edema and endothelial cell loss after intracapsular cataract extraction. Acta Ophthalmol. (Copenh).

[19] Klein R, Klein B.E., Linton K.L., De Mets D.L. (1991). The Beaver Dam Eye Study: visual acuity. Ophthalmology.

[20] Amesbury E.C., Grossberg A.L., Hong D.M, Miller K.M. (2009). Functional visual outcomes of cataract surgery în patients with 20/20 or better preoperative visual acuity. J. Cataract Refract. Sur.

[21] Gills J.P. (2002). Treating astigmatism at the time of cataract surgery. Curr. Opin. Ophthalmol.

[22] Klein B.E., Klein R, Linton K.L., Magli Y.L., Neider M.W. (1990). Assessment of cataracts from photographs în the Beaver Dam Eye Study. Ophthalmology.

[23] Storr-Paulsen A, Norregaard J.C., Ahmed S, Storr-Paulsen T, Pedersen T.H. (2008). Endothelial cell damage after cataract surgery: divide-and-conquer versus phaco-chop technique. J. Cataract Refract. Surg.

[24] Rosado-Adames N, Afshari N.A. (2012). The changing fate of the corneal endothelium în cataract surgery. Curr. Opin. Ophthalmol.

[25] Hayashi K, Manabe S, Yoshimura K, Kondo H (2013). Corneal endothelial damage after cataract surgery în eyes with pseudoexfoliation syndrome. J. Cataract Refract. Surg.

[26] Samudre S.S, Lattanzio Jr. F.A, Williams P.B, Sheppard Jr. J.D. (2004). Comparison of topical steroids for acute anterior uveitis. J. Ocul. Pharmacol. Ther.

[27] Werblin T.P. (1993). Long-term endothelial cell loss following phacoemulsification: model for evaluating endothelial damage after intraocular surgerys. Refract. Corneal Surg.

